# Valorization of Invasive Plant Extracts against the Bispecies Biofilm *Staphylococcus aureus*–*Candida albicans* by a Bioguided Molecular Networking Screening

**DOI:** 10.3390/antibiotics11111595

**Published:** 2022-11-11

**Authors:** Guillaume Hamion, Willy Aucher, Charles Tardif, Julie Miranda, Caroline Rouger, Christine Imbert, Marion Girardot

**Affiliations:** 1 Laboratoire EBI, University of Poitiers, UMR CNRS 7267, F-86000 Poitiers, France; 2 University of Bordeaux, UMR INRAE 1366, Bordeaux INP, OENO, ISVV, F-33140 Villenave d’Ornon, France; 3 Bordeaux Sciences Agro, UMR INRAE 1366, Bordeaux INP, OENO, ISVV, F-33170 Gradignan, France; 4 Bordeaux Metabolome, MetaboHUB, PHENOME-EMPHASIS, Centre INRAE de Nouvelle Aquitaine-Bordeaux, F-33140 Villenave d’Ornon, France

**Keywords:** antibiofilm, invasive plants, natural products, molecular networking, *Staphylococcus aureus*, *Candida albicans*, betulinic acid

## Abstract

Invasive plants efficiently colonize non-native territories, suggesting a great production of bioactive metabolites which could be effective antibiofilm weapons. Our study aimed to look for original molecules able to inhibit bispecies biofilm formed by *S. aureus* and *C. albicans*. Extracts from five invasive macrophytes (*Ludwigia peploides*, *Ludwigia grandiflora*, *Myriophyllum aquaticum*, *Lagarosiphon major* and *Egeria densa*) were prepared and tested in vitro against 24 h old bispecies biofilms using a crystal violet staining (CVS) assay. The activities of the extracts reducing the biofilm total biomass by 50% or more were comparatively analyzed against each microbial species forming the biofilm by flow cytometry (FCM) and scanning electron microscopy. Extracts active against both species were fractionated. Obtained fractions were analyzed by UHPLC-MS/MS and evaluated by the CVS assay. Chemical and biological data were combined into a bioactivity-based molecular networking (BBMN) to identify active compounds. The aerial stem extract of *L. grandiflora* showed the highest antibiofilm activity (>50% inhibition at 50 µg∙mL^−1^). The biological, chemical and BBMN investigations of its fractions highlighted nine ions correlated with the antibiofilm activity. The most correlated compound, identified as betulinic acid (BA), inhibited bispecies biofilms regardless of the three tested couples of strains (ATCC strains: >40% inhibition, clinical isolates: ≈27% inhibition), confirming its antibiofilm interest.

## 1. Introduction

Biofilms are complex structures in which microorganisms belonging to different species can grow, proliferate, interact, communicate and acquire original attributes allowing them to tolerate or resist numerous conventional antimicrobial agents [1].

In human health, biofilms have been the subject of numerous studies over the last few decades to understand the origin of therapeutic failures and relapses in case of infection related to biofilms. Indeed, many fungal and bacterial infections can be associated with a biofilm which develops on a medical device or a biotic surface [1]. The structure and architecture of biofilms are becoming increasingly known, particularly concerning those formed by *Staphylococcus aureus* bacteria and *Candida albicans* yeasts. These two important and ubiquitous species are among the most studied microorganisms because of their frequency of isolation in the case of infections. The retrospective study by He et al. indicated that *C. albicans* was the third most common organism isolated on central venous catheters, after *Acinetobacter* and *Staphylococcus epidermidis*, and was the third most common species causing central line-associated bloodstream infection, just after *Acinetobacter* and *S. aureus*. In this last case, the authors found close prevalence for *S. aureus* (13.1%) and *C. albicans* (12.1%) [2].

*C. albicans* is a commensal species of the human oral cavity, gastrointestinal and reproductive tract. It is also an opportunistic pathogen capable of causing superficial to systemic and hematogenously disseminated candidiasis, depending on the patient’s immune status and other predisposing factors [3,4,5]. In addition, candidiasis is often associated with a biofilm [6]. The Gram-positive bacterium *S. aureus* is a commensal species, as well as an opportunistic pathogen, responsible for superficial to life-threatening diseases, often related to a biofilm [7,8]. *C. albicans* and *S. aureus* share numerous host niches contributing to their frequent coisolation [9]. 

Not surprisingly, numerous cases of mixed *C. albicans*–*S. aureus* infections have been reported, including bloodstream infections [10,11,12]. In this latter case, numerous risk factors have been recently identified, such as a prolonged stay in an intensive care unit, antimicrobial administration and the presence of two or more central venous catheters [11]. 

The propensity of *C. albicans* and *S. aureus* to grow together within interkingdom biofilms is well documented, as well as their respective ability to protect each other from antimicrobial treatments [13]. Thus, the resistance and tolerance inherent in the biofilm lifestyle are worsened by the protection induced by the polymicrobial character of the biofilm, in which communication and interaction processes further strengthen the microorganisms [14,15]. Unfortunately, no treatment is yet available to prevent the severe mortality and morbidity associated to interkingdom biofilms formed by these two infamous species. It is therefore necessary and urgent to search for new and original molecules capable of destroying or inhibiting mixed *C. albicans*–*S. aureus* biofilms, and thus to fight against associated infections [13,14,15].

Invasive alien plants (IAPs) are a source of compounds of interest and could therefore meet this expectation. Invasive alien species are defined as “alien species that reach the final stage of the invasion process and have the capacity to spread [...] with highly detrimental impact in the regions concerned, not only on local biodiversity and on the way ecosystems work, but also on socioeconomic parameters, including animal production and hence animal health, and lastly on public health” [16]. 

According to the International Union for Conservation of Nature (IUCN), invasive alien species are “one of the biggest causes of biodiversity loss and species extinctions” [17]. IAPs often have no natural predators in their new environments and display a high capacity of dispersion and of forming a dense monospecific population entering into competition with the native plants. It is known, for example, that invasive *Ludwigia* species, because of their capacity to rapidly cover the entire surface of a body of water, lead to a modification of the environment that is harmful to the local fauna and flora [18]. 

However, despite these features and the economic, ecological and health-related negative impacts of invasive plants, they also constitute a reservoir of molecules with great potential. Indeed, their ease of adaptation, control of the new habitat and resistance to predators involve their chemical machinery. Some studies mention their capacity to synthesize new or more concentrated allelopathic, defense or antibiotic biochemicals resulting in a different chemical composition than that of native plants [19,20]. 

Thanks to these compounds, some IAPs have previously demonstrated antioxidant, antimicrobial, antiviral, neuroprotective, antiproliferative and cytotoxic, anticholinesterase activities [20]. Among them, several aquatic species have been highlighted. For example, invasive *Ludwigia peploides* (*Onagraceae* family) previously demonstrated antimicrobial, antioxidant and antiproliferative activities [21], while invasive *Ludwigia grandiflora* also demonstrated antibacterial activities against Gram-positive and Gram-negative bacteria [22]. 

Invasive plant extracts are still poorly investigated for their curative activities against polymicrobial biofilms, despite their presumed interesting potentials. Thus, this work aims to demonstrate the interest of invasive plants, in particular aquatic IAPs, in the discovery of compounds active against polymicrobial *S. aureus*–*C. albicans* biofilms. This work focuses on five IAPs registered on the list of plants of concern in France: *Egeria densa*, *L. grandiflora*, *L. peploides*, *Myriophyllum aquaticum* and *Lagarosiphon major*. Thus, this study could provide a new treatment to reinforce the therapeutic arsenal against biofilm and biofilm-related infections.

## 2. Results and Discussion

### 2.1. Plant Extracts

The five plants (*E. densa*, *L. grandiflora*, *L. peploides*, *M. aquaticum* and *L. major)* were identified, sampled and dried. Then, they were successively extracted using four solvents of increasing polarity (MeTHF, EtOAc, EtOH and EtOH/W, respectively). Forty extracts were obtained, and their yields are reported in Table 1. For each plant, the best yield of extraction was obtained with the mixture EtOH/W (2.9–19.7%). The solvents MeTHF and EtOH led to intermediate yields: (1.1–11.2%) and (0.5–11%), respectively. The solvents MeTHF and EtOAc have a close polarity index (4 and 4.4, respectively), which largely explains the low yield associated to EtOAc. Indeed, MeTHF already drained the compounds in this polarity range. Thus, the EtOAc extracts would have a limited interest. The best total yields were obtained with *Ludwigia* species and *M. aquaticum*, especially their leaves. *E. densa* and *L. major* contained fewer compounds extractable by these solvents. 

These results suggested that polar components were present in great quantity in the studied plants. Other studies also observed that, after extraction by solvents of increasing polarity, the highest yields were obtained with the most polar solvents [23]. This observation is not surprising given that numerous primary and secondary metabolites commonly present in plants are polar, including sugars, amino acids, organic acids or most components of the large category of phenolic compounds [24].

### 2.2. Antibiofilm Activities Screening

**Figure 1:**
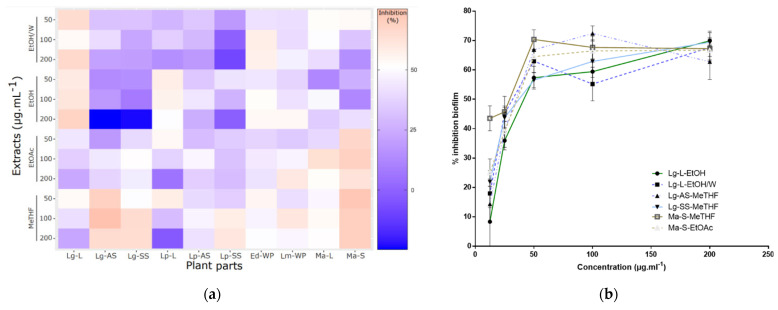
Plant extracts activity against *S. aureus*–*C. albicans* biofilm. Antibiofilm activities were assessed using crystal violet staining assay: (**a**) Heatmap displaying screening results of 40 plant extracts, with in ordinate the solvents of extraction and the final tested concentrations (50, 100, 200 µg∙mL^−1^), and in abscissa the extracted plant part; (**b**) Inhibition curves of the six most active extracts (6.25 to 200 µg∙mL^−1^), expressed in percentage of biofilm inhibition. Abbreviations: Lg (*Ludwigia grandiflora*), Lp (*Ludwigia peploides*), Ed (*Egeria densa*), Lm (*Lagarosiphon major*), Ma (*Myriophyllum aquaticum*), L (leaves), AS (aerial stems), SS (submerged stems with roots), S (stems), WP (whole plants).

The forty extracts were first screened for their activity against bispecies biofilms of *C. albicans* and *S. aureus*, using three concentrations ranging between 50 and 200 µg∙mL^−1^. The ability of these extracts to reduce already-formed 24 h old biofilms was investigated. The activity varied according to the extracts (Figure 1a), but in general, the results suggested that the least polar solvents used (MeTHF and EtOAc) were the most active against biofilms, except for *L. grandiflora* leaves (Lg-L) and *L. peploides* leaves (Lp-L), with MeTHF ones especially being the most active of all. Several studies have already shown the weakly polar nature of many compounds active against biofilms. For example, essential oils have shown a great activity against biofilm adhesion [25]. Some lipids are also known for having antibiofilm activities, especially against mixed *C. albicans* and *S. aureus* biofilms [26]. Concerning *L. grandiflora*, more polar extracts were also highlighted (EtOH and EtOH/W) but they were not obtained from the same part, which suggested that all parts were of interest in this plant: polar compounds from leaves and less polar compounds from AS and SS parts.

Based on these results, completed by a Dunn statistical analysis, six extracts, from *L. grandiflora* leaves (Lg-L), aerial stems (Lg-AS), submerged stems with roots (Lg-SS) and *M. aquaticum* stems (Ma-S), demonstrated a significant antibiofilm activity compared to the nontreated control conditions and were therefore identified as promising: Lg-L-EtOH/W; Lg-L-EtOH; Lg-AS-MeTHF; Lg-SS-MeTHF; Ma-S-EtOAc; Ma-S-MeTHF. Their dose-dependency activity was then shown by testing concentrations above 50 µg∙mL^−1^ (Figure 1b). Due to the closeness of the chromatographic profiles of the Ma-S-MeTHF and Ma-S-EtOAc extracts and the limiting amounts obtained for Ma-S-EtOAc extract, this last one was not further investigated. Thus, the antibiofilm activity of the remaining five selected extracts was then further detailed.

### 2.3. Characterization of Active Extracts

**Figure 2:**
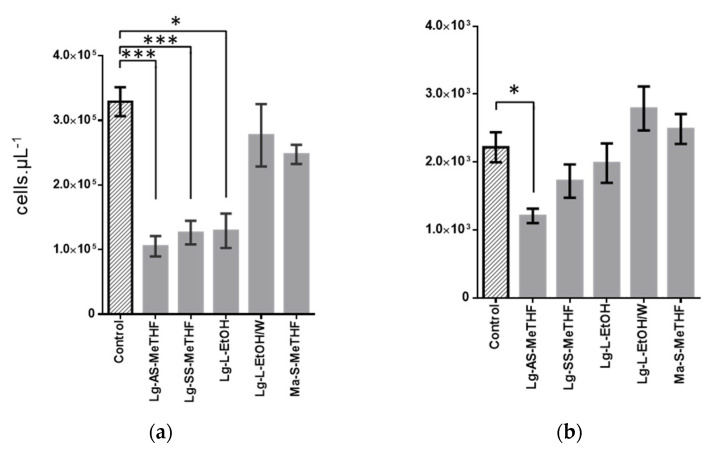
Inhibition activities of crude extracts at 100 µg∙mL^−1^ on 48 h old bispecies biofilm of *C. albicans–S. aureus*: (**a,b**) enumeration of each population in bispecies biofilm (**a**) *S. aureus* and (**b**) *C. albicans* by FCM after 24 h of treatment with the five selected crude extracts, compared with control (DMSO 2% treatment) (error bars in SEM). *p*-value calculated by Dunn’s test were given with *: 0.05 > *p*-value > 0.01; ***: 0.001 > *p*-value.

Through an FCM approach, we evaluated the activity of these five extracts specifically against the bacterial and fungal populations of bispecies biofilms. Indeed, the difference in cell size allowed us to distinguish these two populations, as we previously showed in another bispecies biofilm model [27]. The SYTO9 staining allowed counting the microorganisms obtained after biofilm scraping in order to compare the populations of the control biofilms (DMSO) to those treated by one of the five studied extracts. The number of bacterial cells counted was about 100 times as high as that of the fungal cells (Figure 2a,b). For the five extracts studied, only Lg-AS-MeTHF significantly reduced by a factor of three compared to the control for the bacterial (*p* < 0.001) and by a factor of two for the fungal (*p* < 0.05) population of the bispecies biofilms (Figure 2a,b). The Lg-SS-MeTHF and Lg-L-EtOH extracts also reduced the *S. aureus* population (*p* < 0.05) but had no significant effect on the *C. albicans* one. Finally, the extracts Lg-L-EtOH/W and Ma-S-MeTHF were not active, regardless of the targeted population. Results obtained by CVS and FCM approaches may appear partially diverging. The large difference between the two populations can be explained by the shorter doubling time for bacteria than for yeast in the in vitro condition [28,29]. Moreover, differences between CVS and FCM approaches were not surprising, as these approaches targeted different constituents of the biofilm. The sonication performed before the FCM analyses eliminated the aggregates, resulting in single-cell suspensions. In addition, applied FCM settings allowed to provide quite strict microbial cell counts that excluded other constituents that may be present, such as matrix or cellular fragments and free components. In a different way, the CVS method required several successive washes that may detach the bacteria and yeasts less strongly attached to the biofilm. This method also tagged all constituents of the biofilm [30], thus giving a global view of the biofilm, wider than the microorganism’s count. It is therefore possible that the extracts whose activity was not observed using FCM acted mainly on the matrix.

Addition of propidium iodide (PI) to the microbial suspensions analyzed by FCM allowed to evaluate the effect of the extracts on the cell membrane permeability. PI-labelled cells could be considered dead [27,31]. PI labelling did not reveal any difference between cells from treated biofilms and controls, regardless of the extract studied. This result suggested that the active extracts did not alter the membrane permeability, and thus that all microbial cells present in the cell suspensions analyzed by FCM were alive. This was consistent with the results of SEM observations of the bispecies biofilms treated or not with Lg-AS-MeTHF extract at 50 µg∙mL^−1^ and 100 µg∙mL^−1^. We did not observe any morphological modification of the cells after treatment, neither for bacteria nor for yeasts (Supplementary Materials Figure S1a,b). Unfortunately, this SEM approach did not allow any cell quantification to complete this result.

### 2.4. Bioactive Molecular Networking

In order to identify the largest possible panel of interesting compounds, the extracts both active with CVS and FCM methods most likely to have different compositions were selected for further investigation. As Lg-AS-MeTHF and Lg-SS-MeTHF presented similar HPLC profiles, suggesting a very close composition, the latter was not retained. The Lg-AS-MeTHF and Lg-L-EtOH extracts were finally considered for further investigation. Their fractionation resulted in seven and nine fractions, respectively (Table 2). The strongest antibiofilm activities (bispecies biofilm, CVS method) were associated with the fractions derived from the Lg-AS-MeTHF extract. Two of them exhibited comparable (F5) or even higher (F4) activities than the initial extract. Overall, the most active fractions of this extract were obtained with high percentages of acetonitrile (70–100%), suggesting the presence of moderately or highly apolar compounds. In the case of the Lg-L-EtOH extract, only two fractions (F3′and F4′) exhibited activities comparable to that of the extract, while the others displayed lower activities. The Lg-AS-MeTHF extract was therefore considered the most promising. As fractions and extract were tested at the same concentration, it can be assumed that the fraction 4 was enriched in the compound(s) active against biofilm. A bioactive score was calculated for each fraction and extract. This score considered the variability of biological tests and was defined as the probability for a molecule of being bioactive [32]. These scores allowed composition–activity correlation studies that aimed to highlight compounds supposed to be responsible for, or involved in, the activity of the plant. For this purpose, the chemical profiles of the Lg-AS-MeTHF extract and all its fractions were analyzed, regardless of their bioactive score. Concerning the fractions from the Lg-L-EtOH extract, only the inactive ones (score ≤ 15) were considered so that the analysis was as discriminating as possible. This was made possible since the two selected extracts were prepared from the same plant.

The samples (Lg-AS-MeTHF extract and its fractions 1–7, fractions 1′–2′ and 6′–9′ of Lg-L-EtOH) were analyzed by LC-HRMS^2^ in positive and negative modes in order to cover as many compounds as possible. This approach also facilitated the identification of compounds detected in both active and negative modes. After processing the data on MZmine2, a total of 993 positive precursor ions and 4735 negative ones were detected in the samples for MS^1^. The data obtained in positive and negative ionization were treated separately. Finally, an antibiofilm activity score was assigned to each sample and the area under the curve was calculated for each compound detected. As a result, nine compounds correlated with antibiofilm activity were found, seven in positive mode (LUg1 to LUg7) and two in negative mode (LUgA and B).

**Figure 3:**
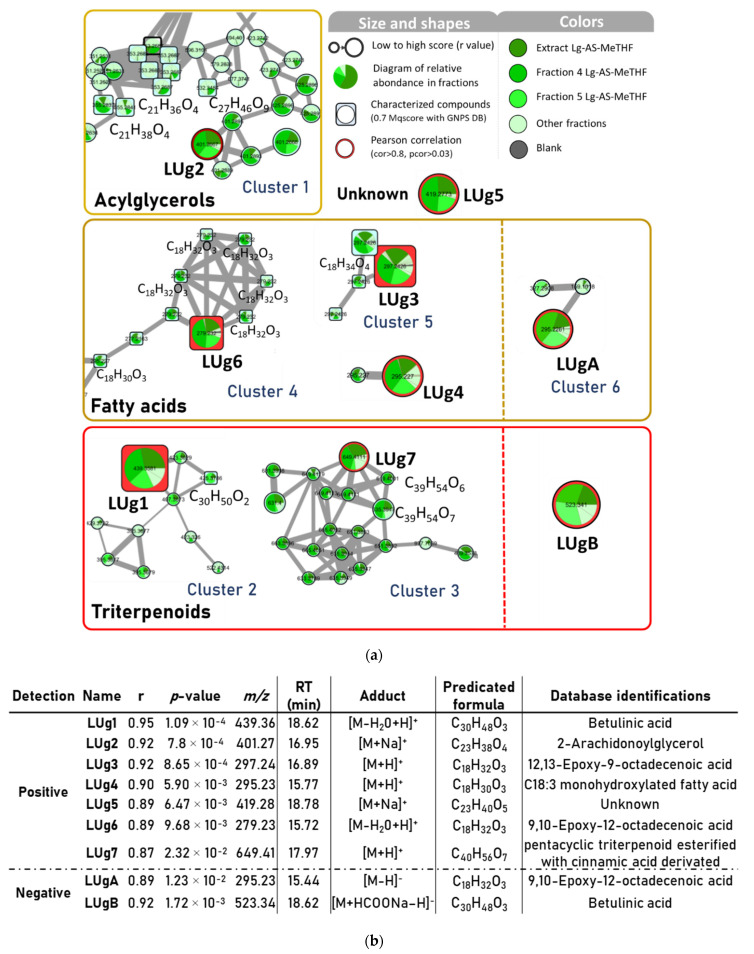
Feature-based molecular networks corresponding to *L. grandiflora* fractions and extracts. (**a**) Molecular networks generated from positive and negative ionization mode, with legend of represented ions. (**b**) List of bioactive correlated ions including correlations scoring (r), *p*-value, *m*/*z* value, retention time (RT), adduct ions detected, predicted chemical formula and identification predicted by databases (GNPS, MetFrag).

Molecular networks were constructed to partially or completely elucidate the structure of these nine ions, represented with their correlation scores and their identification from GNPS databases and/or from manual annotation using in silico fragmentation software MetFrag (Figure 3).

Of the seven positive ions correlated with the bioactivity (Figure 3a), the most correlated one called LUg1 (correlation score r at 0.95, with *p*-value at 1.1 × 10^−4^) was detected at *m*/*z* 439.3581 and a retention time (RT) of 18.62 min. GNPS databases identified it as an [M−H_2_O+H]^+^ ion with the calculated chemical formula C_30_H_47_O_2_ corresponding to the pentacyclic triterpenoid betulinic acid (BA). In the same network as BA, another triterpenoid matched with the database search: the betulin (C_30_H_50_O_2_), displaying a structure close to BA with a primary alcohol instead of carboxylic acid function on the 28th carbon (Figure S2). BA was available as commercial standard (Sigma-Aldrich, Saint Louis, MO, USA), and in order to confirm its identification, the HPLC-UV (210 nm) and targeted HPLC-MS/MS profiles of F4 and the Lg-AS-MeTHF extract were compared with the standard. Results showed a peak at the same retention time (Figure S3a) and a similar MS/MS fragmentation profile (*m*/*z* fragments and their relative intensity) (Figure S3b), which supported the BA identification hypothesis.

Two other putative bioactive compounds highly correlated, LUg3 (*m*/*z* 297.2426 at 16.89 min) and LUg6 (*m*/*z* 279.2319 at 15.72 min), were identified in the GNPS database as epoxidized fatty acids, respectively, as [M+H]^+^ of 12,13-epoxy-9-octadecenoic acid and [M−H_2_O+H]^+^ 9,10-epoxy-12-octadecenoic acid. LUg3 was networked with three ions with the same *m*/*z* of 297.2426, including a hypothetical stereoisomer (RT at 17.1 min), [M−H_2_O+H]^+^ 9,10-dihydroxy-12-octadecenoic acid (RT at 16.95 min) and [M+H]^+^ 9,10-epoxy-12-octadecenoic acid (RT at 13.63 min) (Table S1). Lug6 was also networked with identified compounds on GNPS: as probably a stereoisomer ([M−H_2_O+H]^+^ 9,10-epoxy-12-octadecenoic acid, RT at 15.44 min), five closed structures of linolenic acid ([M+H]^+^ *m*/*z* 279.2320 at 17.97, 18.1, 18.35, 18.82 min), [M−H_2_O+H]^+^ 9-hydroxy-10,12,15-octadecatrienoic acid (*m*/*z* 277.2163 at 16.31 min) and two putative isomers of [M+H]^+^ 9-oxo-10,12-octadecadienoic acid (*m*/*z* 295.2270 at 16.14 and 16.33 min). These networks of fatty acids and derivatives confirmed the nature of the LUg3 and LUg6 structures. However, the complexity of their determination and difficulties for synthetizing standards would require the isolation and investigation of their absolute structure.

The remaining bioactivity correlated ions were not identified within the GNPS database but through in silico fragmentation, either by direct comparison on MetFrag with their MS/MS spectra and/or by comparing the MS/MS spectra of their networked ions, thus helping to improve their identifications. For LUg2 (*m*/*z* 401.2667 at 16.95 min), with the chemical formula C_23_H_38_O_4_Na (Δ*_m_*_/*z*_ = 1.169 ppm), the postanalysis attributed 37 out of 59 similar fragments on its MS/MS spectrum with theoretical MS/MS spectrum of [M+Na]^+^ 2-arachidonoylglycerol (C_23_H_38_O_4_). On the LUg2 cluster, five putative stereoisomers of [M+H]^+^ monolinolenin (*m*/*z* 353.2687 at 12.63, 13.07, 13.33, 14.53, 14.64 min), [M+H]^+^ 1-linoleoylglycerol (*m*/*z* 355.2837 at 14.08 min) and [M+NH_4_]^+^ 9,12,15-octadecatrienoic acid, 3-(hexopyranosyloxy)-2-hydroxypropyl ester (*m*/*z* 532.3484 at 14.61 min) were identified with the GNPS database (Supplementary Materials Table S1). All these compounds were composed of a glycerol part esterified with a long chain unsaturated fatty acid. These assumptions are consistent with the identification of LUg2 as 2-arachidonoylglycerol (Figure S2).

LUg4 (*m*/*z* 295.2269 at 15.77 min) was found to be related to the in silico fragmentation of [M+H]^+^ 17-hydroxyoctadeca-9,11,13-trienoic acid (47 out of 69 fragments in MS/MS spectrum). In parallel, a negative ion at the same retention time was detected (*m*/*z* 293.2124 at 15.76 min) (Figure 3b), and the MS/MS comparison gave [M−H]^−^ 2-hydroxylinolenic acid (12/31 similar fragments), implementing the hypothesis that LUg4 was a C18:3 monohydroxylated fatty acid. 

In the same way, LUg7 (*m*/*z* 649.4100 at 17.97 min) had a high number of similar peaks, with an in silico spectrum of eucalyptic acid (52 matches/79 peaks), 3-*O*-feruloyl-2-hydroxy-12-ursen-28-oic acid (52/79) and 11-hydroxy-10-{[3-(3-hydroxy-4-methoxyphenyl)prop-2-enoyl]oxy}-1,2,6a,6b,9,9,12a-heptamethyl-1,2,3,4,4a,5,6,6a,6b,7,8,8a,9,10,11,12,12a,12b,13,14b-icosahydropicene-4-carboxylic acid (58/79). These three molecules have C_40_H_56_O_7_ as a molecular formula and consist of a pentacyclic triterpenic part esterified with a phenolic acid (ferulic or isoferulic acid). Two networked compounds with LUg7 were assimilated to similar structures: *m*/*z* 619.4001 at 17.75 min as 2-*O*-*p*-coumaroyl alphitolic acid (45/60) (C_39_H_54_O_6_) and *m*/*z* 635.3947 at 16.98 min as 3-caffeoyloxy-2-hydroxyurs-12-en-28-oic acid (38/58) (C_39_H_54_O_7_). Moreover, the negative ion of LUg7 at *m*/*z* 647.3964 showed spectral similarities with the 3-alpha-*O*-*trans*-feruloyl-2-alpha-hydroxy-12-ursen-28-oic acid and the *trans*-3-feruloylcorosolic acid (10/23 common fragments, molecular formula C_40_H_56_O_7_) (Figure 3a). All these similarities converged towards the hypothesis of a pentacyclic triterpenic structure esterified with a cinnamic acid derivative for LUg7. Seven other networked compounds matched with the GNPS databases and were identified as triterpenoïds (Table S1).

LUg5 (*m*/*z* 419.2773 at 18.78 min) showed a low number of peaks in the MS/MS spectrum, but an intense ion at *m*/*z* 207.0995 (100% relative intensity) and another main one at *m*/*z* 335.2196 (7.5%). The calculated raw formula for the parent ion was C_23_H_40_O_5_Na (Δ*_m_*_/*z*_ = 0.965 ppm). Comparisons with databases gave no similarity with known natural compounds, suggesting the presence of a new compound. 

Finally, the last two compounds correlated in the molecular network with the negative ionisation mode, LUgA (*m*/*z* 295.2281 at 15.44 min) and LUgB (*m*/*z* 523.3410 at 18.62 min), shown in Figure 3a, which exhibited similarities with the ions already described. LUgA was the [M−H]^−^ adduct of LUg6. Its MS^1^ calculated molecular formula (C_18_H_31_O_3_) and the in silico comparison of its MS^2^ spectrum resulted in its identification as [M−H]^−^ 9,10-epoxy-12-octadecenoic acid (12/31 common fragments). LUgB showed the same retention time as LUg1 (BA), with a major MS^2^ peak at *m*/*z* 455.3535, assuming that LUgB is the [M+HCOONa–H]^−^ adduct of BA.

To summarize, the activity of Lg-AS-MeTHF is correlated with two families of molecules. The first one is lipids including acylglycerols and derivatives of fatty acids, especially hydroxylated and epoxidized derivatives. Epoxidized and hydroxylated fatty acids are often found in plants and are notably part of oxylipins biosynthesis (enzymatically oxygenated fatty acids), metabolites involved in intra/intercellular communication in plants [33]. The presence of free fatty acids as linoleic, linolenic and arachidonic acids was also confirmed in the close species *Ludwigia octovalvis* [34]. Several studies highlighted the antibiofilm activities of fatty acids such as linoleic acid and their derivatives on Gram-positive bacteria (including *S. aureus*) [35,36]. Moreover, fatty acids would have effects on intraspecies microbial communication as *C. albicans* quorum sensing. Indeed, they can, for example, mimic the effect of farnesol, an important signal molecule secreted by *Candida* yeasts, making the study of fatty acids interesting to target the yeast hyphal form [37,38]. The second chemical family of interest concerns pentacyclic triterpenes such as betulinic acid, a secondary metabolite of many plants involved in the biosynthesis of saponins, natural surfactant biomolecules [39]. BA and betulin were also identified in the *Ludwigia adscendens* extract [40]. Several pentacyclic triterpenoids, such as glycyrrhetinic acid, ursolic acid and BA, have been shown to display antibiofilm effects against Gram-negative bacteria *Acinetobacter baumannii* and *Pseudomonas aeruginosa* [41]; an activity of BA and several derivatives was also described on the *S. aureus* biofilm [42].

Among the nine correlated ions, BA appeared to be the best candidate molecule to be involved in the antibiofilm activity of the *L. grandiflora* extract. Its activity was investigated on the bispecies biofilm to confirm this hypothesis using commercial pure BA.

### 2.5. Antibiofilm Activity and Quantification of Betulinic Acid

**Figure 4:**
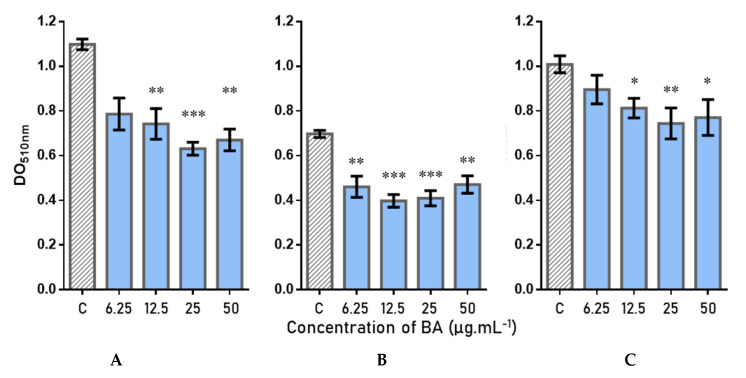
Betulinic acid (BA) activities on total biomass (CVS assay) of bispecies biofilm strains of *C. albicans*–*S. aureus* (error bars in SEM): (**A**) *C. albicans* ATCC 28367–*S. aureus* ATCC 29213; (**B**) *C. albicans* ATCC MYA-2876–*S. aureus* ATCC 6538; (**C**) *C. albicans* Aca1–*S. aureus* SCO4. *p*-values calculated by Dunn’s test were given with *: 0.05 > *p*-value > 0.01; **: 0.01 > *p*-value > 0.001; ***: 0.001 > *p*-value.

BA effects on 24 h mature biofilms were assayed using the same protocol as for plant extracts, with a CVS measurement of the total biomass after treatment. Different concentrations, ranging from 6.25 to 50 µg∙mL^−1^ (13.7 to 109.5 µM) (concentrations below BA’s solubility limit), were tested on three couples of microorganisms: two were constituted of reference strains (Couple A: *C. albicans* ATCC 28367–*S. aureus* ATCC 29213; Couple B: *C. albicans* ATCC MYA2876–*S. aureus* ATCC 6538) and one of clinical isolates (Couple C: *C. albicans* Aca1–*S. aureus* SCO4). The results, shown in Figure 4, highlighted a significant inhibition of the biofilm of at least 12.5 µg∙mL^−1^ for the reference strains (A and B). Biofilm inhibition reached up to 42% at 25 µg∙mL^−1^ (55 µM) for pair A, with a loss of activity from 12.5 µg∙mL^−1^ (27.4 µM). For couple B, BA showed activity at lower concentrations than for couple A, with still 34% inhibition at 6.25 µg∙mL^−1^, but showed similar activity at 25 µg∙mL^−1^ (41–43% inhibition). For the clinical strains pair, we observed a lower inhibition than for the collection couples, with a maximum obtained inhibition of 27% at 25 µg∙mL^−1^. 

Thus, these results showed an activity against the bispecies biofilm *C. albicans*–*S. aureus*, regardless of the tested couples (with a slightly lower activity for the clinical strains, generally more resistant to treatments), highlighting the nonstrain dependence and the interest of this compound.

BA was quantified by HPLC-UV within F4, F5 (most active fractions) and the Lg-AS-MeTHF extract in order to investigate the activity/BA quantity relationship (Figure S4). Analyses suggested that 1 mg of F4 and F5 contained 169.31 µg and 24.79 µg of BA, respectively, and that 1 mg of the extract contained 23.73 µg of BA. Thus, F4 has been strongly enriched in BA. The 64% and 50% of biofilm reduction induced by F4 and F5 at 50 µg∙mL^−1^ corresponded to a concentration of 8.5 and 1.2 µg∙mL^−1^ of BA in contact with the biofilm. BA alone did not demonstrate such high activity on the bispecies biofilm before, and efficient concentrations reached around 25 µg∙mL^−1^ BA (43% biofilm inhibition), which suggested that BA was not the only compound responsible for the activity. Nevertheless, the highest concentration of BA in F4 compared to F5 seems to be correlated with a higher activity. A study of the other correlated compounds would allow to check the potential synergistic effect between the different active ingredients contained in the extracts of *L. grandiflora*.

BA is fairly well known, but its antibiofilm potential requires our full attention. A wide variety of actions has already been associated with BA (anticancer, anti-inflammatory, anti-HIV, antimalarial and anthelmintic activities), yet no cytotoxicity has been reported on healthy cells [43]. Some studies have described the mechanism of action of BA, particularly on mammalian myeloma cells. BA induced the apoptosis of cells by targeting mitochondria, generating an increase of reactive oxygen species in cytosol, and consequently an oxidative stress [44]. For all these applications, BA is still largely investigated in different studies, including its improvement of its bioavailability, due to its low solubility in aqueous medium that limiting its applications. One approach under consideration is its incorporation in nanoparticles, that permits to enhance its concentration and control its release during treatment [45]. The large applications of BA and actual studies of this molecule make it a promising treatment in human health.

## 3. Materials and Methods

### 3.1. Plant Material

Five aquatic IAPs: *E. densa*, *L. grandiflora*, *L. peploides*, *L. major* and *M. aquaticum* were collected in July 2020 in west region of France (Nouvelle-Aquitaine) (GPS localization: *E. densa* (46.569233, 0.640664); *L. grandiflora* (46.645098, 0.584291); *L. peploides* (46.910908, 0.247578); *L. major* (46.557362, 0.409080); *M. aquaticum* (45.645026, −0.053613)). Botanical identification was performed by the Conservatoire Botanique National Sud-Atlantique (CBNSA). Samples were washed in water baths and air-dried for one week. A voucher specimen of each plant was deposited at the Herbarium of the School of Pharmacy at the University of Poitiers (France).

### 3.2. Chemicals and Reagents

LC-MS grade acetonitrile (ACN) and methanol (Fisher Scientific, Waltham, MA, USA) were used for the UHPLC analysis. Extractions and fractionation were performed with 2-methyltetrahydrofuran (MeTHF), ethyl acetate (EtOAc), ethanol (EtOH), isopropanol (IPA) and cyclohexane (CHX) (E. Merck, Darmstadt, Germany). Deionized water (W) was purified by Milli-Q system (Millipore, Burlington, MA, USA). Betulinic acid analytical grade was purchased from Sigma–Aldrich Chemical Corporation (Saint Louis, MO, USA).

### 3.3. Extraction and Fractionation

Whole plants (WP) or parts of plants (stems (S) and leaves (L) for *M. aquaticum* leaves, aerial stems (AS) and submerged stems with roots (SS) for *L. grandiflora* and *L. peploides*) were reduced to a powder and 20 g were extracted by maceration assisted by sonication for 1 h at room temperature. Four solvents of increasing polarity were used successively on the same sample: MeTHF, EtOAc, EtOH, EtOH-W (1:1 *v*/*v*). After filtration by using a Büchner funnel, the extracts were evaporated under low pressure at 40 °C. A supplementary step of freeze-drying was added for MeTHF and EtOH-W extracts. 

The most active extracts against bispecies biofilm (600 mg of EtOH leaves extract from *L. peploides* and 250 mg of MeTHF aerial stem extract from *L. grandiflora*) were fractionated by using flash chromatography (Puriflash^®^ 4250 from Interchim (Montluçon, France) equipped with a diode array detector) on prepacked C18 columns (C18-HP 30 µm, 51 g for EtOH extract and 32 g for MeTHF extract, Interchim). Samples were solubilized in MeOH to perform a liquid loading, and compounds elution was monitored using UV detection at 220 and 265 nm. Compounds were eluted at 10 mL∙min^−1^ with ACN/H_2_O (5:95 to 30:70 in 30 min and then 30:70 to 100:0 in 5 min for EtOH extract; 5:95 to 60:40 in 15 min and then 60:40 to 100:0 in 5 min for MeTHF extract), and finally eluted for 30 min using, successively, 100% ACN, 100% IPA and 100% CHX to afford nine and seven fractions from EtOH and MeTHF extracts, respectively. The fractions were evaporated under low pressure at 40 °C and/or by lyophilization. Dried fractions and extracts were stored at −80 °C.

### 3.4. HPLC Analysis

All extracts and fractions were analyzed on DIONEX UltiMate 3000 UHPLC (Thermo Fisher scientific, Waltham, MA, USA) with a diode array detector (UHPLC-DAD) on a C18 analytical column (DIONEX, C18, 5 µm, 120 Å, 4.6 mm × 250 mm Acclaim^®^) protected by a Phenomenex^®^ SecurityGuard (Torrance, CA, USA). The elution was performed with ACN/H_2_O gradient complemented with 0.1% of trifluoroacetic acid (5:95 to 100:0 during 40 min and then 100:0 for 10 min, 0.8 mL∙min^−1^). The column oven temperature was set at 25 °C. UV detection was monitored at 210, 220 and 265 nm. Samples were injected from 1 to 10 mg·mL^−1^ in MeOH after centrifugation.

### 3.5. Organisms

Three reference strains of *C. albicans* and three reference strains of *S. aureus* were used: *C. albicans* ATCC^®^ 28367™, *C. albicans* ATCC^®^ MYA-2876™, *C. albicans* Aca1 (isolate recovered from venous catheter), *S. aureus* ATCC^®^ 29213™, *S. aureus* ATCC^®^ 6538 ™, *S. aureus* SC04 (isolate recovered from human lungs). *C. albicans* and *S. aureus* were grown for 48 h on Sabouraud glucose with chloramphenicol (0.05 g·L^−1^) (SGC) (Sigma-Aldrich, Saint Louis, MO, USA) or brain heart infusion (BHI) (BD DifcoTM, Sparks, MD, USA) agar plates at 37 °C, respectively. Prior to biofilm assays, each strain was cultured in liquid BHI medium at 37 °C overnight, with agitation at 80 rpm only for *S. aureus*.

### 3.6. Biofilm Studies

#### 3.6.1. Biofilm Growth

An amount of 25 mL of previously prepared BHI liquid cultures were centrifuged at 5000 g for 5 min. The pellet was washed with 10 mL of Phosphate Buffer Saline (PBS) (GIBCO, New York, NY, USA) and centrifuged again in the same condition. Cell concentration was determined by absorbance measurement at 600 nm for *S. aureus* and by using the previously determined equation:

1.4 DO_600nm_ = 23 × 10^9^ CFU/mL (1)

A direct counting with a Fast-Read 102^®^ counting chamber (Biosigma, Cantarana, Italy) was performed for cell concentration determination of *C. albicans*. 

Single- and dual-species biofilms were cultured in 96-well polystyrene nontreated microtiter plates (Costar, Corning, NY, USA). For single-species biofilms, 200 µL of cultures at 10^6^ CFU∙mL^−1^ for *S. aureus* and 10^6^ cell∙mL^−1^ for *C. albicans* were inoculated in each well. For dual-species biofilms, 100 µL of each suspension at 10^6^ CFU∙mL^−1^/10^6^ cell∙mL^−1^ were inoculated for a 1:1 ratio. After incubation at 37 °C for 2 h, culture medium was removed to eliminate nonadherent cells, and 200 µL of fresh BHI medium was added. After 24 h of total incubation at 37 °C, supernatants were discarded and biofilms were washed once with PBS. An amount of 196 µL of fresh medium and 4 µL of DMSO (control condition) or extract/fraction suspended at 10 mg∙mL^−1^ in DMSO was added. Wells without treatment were preserved (negative control). Plates were incubated for 24 h at 37 °C.

#### 3.6.2. Crystal Violet Staining (CVS) Assay 

After 24 h of incubation, wells were washed with 200 µL of PBS. An amount of 200 µL of MeOH was then added in each well and left in contact during 10 min. After removing MeOH, 200 µL of crystal violet (0.3% in demineralized water) was added and the plates were incubated at room temperature for 5 min. Excesses of crystal violet were washed with demineralized water and 200 µL of acetic acid 33% was added and left in contact during 15 min under agitation (150 rpm). The absorbance was measured at 510 nm (Infinite M Plex absorbance reader, TECAN, Zürich, Switzerland).

The percentage of inhibition (*I*) of each sample was calculated with Formula (2) by comparing with intraplate controls (DMSO 2%).

(2) $$I\;\left(\%\right)=\left(\frac{\mathrm{DO}510_{control}-\;\mathrm{DO}510_{x}}{\mathrm{DO}510_{control}}\right)\times 100$$

The inhibitory percentages and the concentration that inhibited 50% of the biofilm formation (IC_50_) were determined for each tested sample by constructing a dose–response curve and selecting the closest tested concentration value above or equal to 50% inhibition.

#### 3.6.3. Flow Cytometry (FCM) Assay 

According to the adapted protocol of Kerstens et al. [46], after 24 h of incubation, each well was washed with 200 µL of PBS to remove residual nonadherent cells. An amount of 50 µL of PBS previously filtrated at 0.1 µm was added in each well and the biofilms were scratched vigorously with a sterile folded cone before pipetting. All the obtained suspensions were diluted one tenth in filtrated PBS, followed by 10 min of sonication and 30 s of vortex. Suspensions were double stained with 1 to 2 µL of 334 µM SYTO 9 (S9) and 1 µL of 2 mM propidium iodide (PI) (LIVE/DEAD™ Viability/Cytotoxicity Kit, Invitrogen, Carlsbad, CA, USA). Measurements were performed with a CytoFLEX flow cytometer (Beckman Coulter, Brea, CA, USA) equipped with a blue diode laser (excitation 488 nm) and a violet diode laser (excitation 405 nm) managed by CytExpert 2.0.0.153 software (Beckman Coulter) (SYTO 9 excitation filter, 525/40 nm; PI excitation filter, 610/20 nm). A compensation matrix was defined using unstained and single-stained heat-killed biofilm suspension (60 °C during 15 min) prior to sample measurements.

#### 3.6.4. Cryo-Scanning Electron Microscopy

Bispecies biofilms were grown as described above with slight differences: 600 µL of each strain was inoculated at 1 × 10^6^ cells∙mL^−1^ on sterile polycarbonate coupons (diameter: 13 mm, thickness: BIOFOULING 3514 mm; BioSurface Technologies Corporation, Bozeman, MO, USA) deposed in 24-well microplates. After 2 h, the medium was substituted with 1.2 mL fresh BHI. After 24 h, extracts or 2% DMSO were added and the biofilm grew for an additional 24 h. Coupons were then recovered and dried few minutes before they were frozen with liquid nitrogen (Leica EM VCM Vacuum Cryo Manipulation system), sublimed and coated with platinum (Leica EM ACE600 High Vacuum Coater). Samples were then observed with a FEI Teneo Volume Scope (FEI Company, Hillsboro, OR, USA).

### 3.7. Molecular Networking

#### 3.7.1. Data-Dependent LC-ESI-HRMS^2^ Analyses

Selected extracts and fractions of *L. grandiflora* were suspended in MeOH at 1 mg∙mL^−1^. After vortex and sonication, samples were filtrated at 0.2 µm. The sequence was prepared by injecting the samples randomly, with a quality control sample every 10 samples analyzed, consisting in the mixture of an equal volume of all samples. A blank control with 100% MeOH was prepared and analyzed before and after the sample list. The UHPLC was performed on a Vanquish system (Thermo Fischer Scientific, Les Ulis, France) using a reverse-phase column (Zorbax RRHD SB-C18 1.8 µm, 2.1 mm × 100 mm, Agilent Technologies, Les Ulis, France) with a similar method to HPLC analysis, adapted for UHPLC (5:95 to 100:0 ratio of ACN/W gradient during 20 min and then 4 min at 100:0, 0.4 mL∙min^−1^). MilliQ W and ACN were acidified by 0.1% of formic acid. One µL per sample was injected. Column temperature was set at 30 °C.

Mass spectra were acquired in a Q-Exactive Plus^TM^ mass spectrometer (Thermo Fischer Scientific) equipped with a heated electrospray ionization (HESI-II) probe. Acquisition was performed using both positive and negative ionization modes, setting spray voltage at 3.7 kV and 2.8 kV, respectively, capillary temperature at 310 °C and probe heater temperature at 280 °C. The MS^1^ scan range was 150–1200 *m*/*z* with a resolution at 70,000 (full width at half maximum (FWHM) at *m*/*z* 200). Each full MS scan was followed by data-dependent acquisitions (DDA) selecting the 5 most intense ions and acquiring MS^2^ between 50 and 1200 *m*/*z*, with a resolution of 17,500 and a normalized collision energy (NCE) of 20%, 35% and 50%. Data were acquired in centroid format.

#### 3.7.2. MZmine 2 Data Preprocessing Parameters

The spectral features detection of MS/MS data was performed on MZmine 2.53 [47]. Each file of MS^2^ analysis was imported in RAW format. Positive and negative ionization modes data were treated independently. Positive acquisitions were adjusted with asymmetric baseline corrector to 1E7. Peaks detection was set at 2E6 in MS^1^ level and 0 in MS^2^ level. Chromatograms were built for the detected ions with the “ADAP chromatogram builder” algorithm, with a minimum group size of 4 scans, a group intensity threshold of 2E6, a minimum highest intensity of 6E6 and an *m*/*z* tolerance of 0.0015 or 5 ppm. Peaks deconvolution was applied with the “local minimum search” algorithm with the following parameters: chromatographic threshold of 10%, search minimum in RT range of 0.5 min, minimum relative height of 10%, minimum absolute height of 6E6, min ratio of peak top/edge of 1 and peak duration range from 0 to 0.5 min. MS^2^ scan pairing was set at the *m*/*z* range of 0.02 Da and the RT range of 0.1 min. Isotopic peaks grouper was applied with an *m*/*z* tolerance of 0.0015 or 5 ppm, with an RT tolerance of 0.2 min and a maximum charge of 2. Representative isotope was set on most intense. Features alignment step with Join aligner was performed with an *m*/*z* tolerance at 0.0015 or 5 ppm, with a weight for *m*/*z* of 75% and a weight for RT of 25%. The RT tolerance was set at 0.2 min. A final gap-filling step was performed with 10% intensity tolerance, 0.0015 *m*/*z* or 5 ppm tolerance, 0.2 min RT tolerance and with an RT correction. Exportation of data generated *.csv and *.mgf files with MS^2^ data and MS^1^ peaks area integration. The same parameters were applied to negative ionization data, with the exception of noise level, which was set to 5E5 for MS^1^ peaks detection. 

#### 3.7.3. Molecular Networks Analysis

Molecular networks were created using Global Natural Products Social molecular networking (GNPS, http://gnps.ucsd.edu, accessed on 21 October 2022). The MGF and CSV files of processed data on MZmine 2 were uploaded on GNPS. Files were used to generate an MS/MS molecular network using the GNPS Feature-Based Molecular Networking workflow [48]. The precursor ion mass tolerance and the product ion mass tolerance were set to 0.02 Da. Networks were generated using 5 minimum matched peaks and a cosine score of 0.6. The library search options were set to minimum 5 matched peaks and a score threshold of 0.6 without search of analogs. In complement to GNPS databases, in silico fragmentation software MetFrag was used with several molecular databases (Pubchem, Human Metabolome Database (HMDB), Coconut).

Predictions of active compounds were realized with workflow from Nothias et al. [31] from an R-based Jupyter notebook available on GitHub, https://github.com/DorresteinLaboratory/Bioactive_Molecular_Networks (accessed on 21 October 2022). Briefly, bioactivity scores of antibiofilm inhibition at 50 µg∙mL^−1^ from CVS tests were calculated to optimize disparity between samples with the Formula (3).

(3) $$Bioactive\;score=\frac{\left(I-SD\right)\times 100}{\begin{array}{c} I_{max}-SD_{Imax} \end{array}}$$

where the standard deviation (*SD*) from each condition was subtracted to the percentage of inhibition (*I*). A bioactive score of 100 was attributed to the most active sample (*I_max_*). For the other samples, scores were attributed proportionally relative to inhibition (%). A score of 0 was assigned to the samples with negative values.

Scores were added on the spectral features table obtained with MZmine 2 workflow. The Jupyter notebook applied 3 steps of analysis: (i) normalization of TIC intensity, (ii) calculation of Pearson correlation and its significance (*p*-value) between features and bioactivity scores and (iii) Bonferroni correction. Results were imported as a table in Cytoscape 3.8.2 [49] on the molecular network data from GNPS. Compounds which were clustered with correlated compounds, i.e., displaying MS/MS spectral similarities, were also studied to identify or confirm their molecular family. *p*-value and correlation value thresholds were respectively defined as ≤0.05 and ≥0.85.

### 3.8. Annotation and Quantification of Betulinic Acid

In order to confirm the identification and to quantify BA in the MeTHF aerial stems extract of *L. grandiflora* and its F4 and F5 fractions, commercial standard of BA was analyzed by HPLC-DAD and HPLC-MS/MS. HPLC-DAD analysis was performed with the same method as described before, adapted with a flow at 0.5 mL∙min^−1^. Ten µL of samples were injected, with BA at 0.5 mg∙mL^−1^ in MeOH, and crude extract and fractions at 1 mg∙mL^−1^ in MeOH. Each sample was injected separately and mixed in solution (extract or fractions mixed with BA). To quantify BA, 7 dilutions (0.5 to 0.01 mg∙mL^−1^ in MeOH) of the standard were injected to realize a standard curve. UV detection was monitored at 210 nm, corresponding to the maximal absorbance of BA, as described in Zhao, Yan and Cao [50]. HPLC-MS/MS analysis was performed on a Waters system equipped with a time-of-flight XEVO™ G2 Q-TOF analyzer (Waters Corporation, Milford, MA, USA) with an ESI source in positive mode with the same chromatographic method as HPLC-DAD, and a 5 µL injection of the samples. MS^2^ targeted acquisition was set at *m*/*z* 439.36 and 457.36, corresponding, respectively, to the [M+H−H_2_O]^+^ and [M+H]^+^ adducts. Source was set at 120 °C at 3.7 eV, collision energy at 40 eV and acquisition range at *m*/*z* 50–1000 with centroid mode.

Data were analyzed using MassLynxTM software (V4.1, 2013) from Waters.

### 3.9. Statistical Analyses

All biological experiments were performed at least three times with triplicate for each condition. 

The Kruskal–Wallis test with Dunn’s multiple comparisons test was applied to determine the statistical significance between obtained measures with treated biofilms and controls, using GraphPad Prism^®^ version 6.01 (GraphPad Software Inc, San Diego, CA, USA). 

PCA and OPLS-DA tests on MZmine2 data including QCs were realized to check the method performance of LC-MS acquiring data, on SIMCA 14.1 software (Sartorius, Goettingen, Germany).

## 4. Conclusions

The aerial stem extract of *L. grandiflora* demonstrated the highest activity against the bispecies biofilm *C. albicans*–*S. aureus* among the 40 extracts prepared from five aquatic invasive plant species. Biochemometric studies have highlighted several families of compounds potentially responsible for this activity, including fatty acids and their hydroxylated and epoxidated derivatives, monoacylglycerols and pentacyclic triterpenoids. Antibiofilm tests confirmed the betulinic acid activity, a pentacyclic lupane-type triterpenoid, which opens interesting perspectives for the research of new treatment to fight multispecies biofilms. Its mechanism of action must now be further investigated and characterized, and further studies are needed to increase its solubility and bioavailability. Its original structure opens interesting perspectives for possible combinations with already available conventional antibiotic and/or antifungal agents that could be complementary by both destructuring the biofilm and killing microbial cells. Moreover, the isolation of the other compounds correlated to the antibiofilm activity in the pure state will be necessary to complete this study of the *L. grandiflora* extract antibiofilm potential. Finally, this work confirmed the interest of invasive aquatic plants in the discovery of compounds active against polymicrobial biofilms and encourages their further studies.

## Figures and Tables

**Table 1:** Extraction yields of different invasive plant parts, obtained with four solvents.

Plants and Parts ^2^ Extracted		Extraction Solvents				Total per Plant	
		MeTHF ^1^	EtOAc	EtOH	EtOH/W		
		Yield (%)				Yield (%)	Weight (mg)
*E. densa*	WP	1.09	0.58	1.39	4.59	7.67	2142
*L. major*	WP	1.19	0.06	0.56	2.89	4.69	940
*M. aquaticum*	S	4.85	0.95	8.52	19.30	33.64	6731
	L	6.59	1.05	11.09	19.75	38.49	7701
*L. peploides*	AS	2.54	0.30	3.41	16.09	22.37	4476
	SS	2.18	0.19	3.07	11.42	16.87	3376
	L	11.21	0.16	6.44	19.62	37.42	7496
*L. grandiflora*	AS	2.73	0.12	2.84	13.20	18.90	3783
	SS	2.35	0.13	1.48	8.63	12.60	2522
	L	5.33	0.56	6.92	16.50	29.32	5873

^1^ The used solvents are listed in running order: 2-methyltetrahydrofuran (MeTHF), ethyl acetate (EtOAc), ethanol (EtOH) and ethanol/water 50:50 (EtOH/W). ^2^ Abbreviation of parts: WP: whole plant, S: stems, L: leaves, AS: aerial stems, SS: submerged stems with roots.

**Table 2:** Yields, antibiofilm activity and bioactivity scores calculated for *L. grandiflora* Lg-AS-MeTHF and Lg-L-EtOH fractions and crude extracts.

		Yield	Activity (CVS, 50 µg∙mL^−1^)		Bioactive Score
		(%)	Inhibition (%)	SD (%)	
Lg-AS-MeTHF (238 mg)	F1	7.11	0	20.03	0
	F2	1.50	0	27.50	0
	F3	2.59	30.82	9.89	37
	F4	1.10	63.96	6.89	100
	F5	1.77	50.83	8.16	74
	F6	1.87	39.19	13.63	44
	F7	14.54	25.61	12.55	23
	Extract		53.88	7.96	80
Lg-L-EtOH (600 mg)	F1′	15.71	0	21.20	0
	F2′	1.84	10.39	18.70	0
	F3′	9.13	33.85	13.96	34
	F4′	4.14	39.26	9.52	52
	F5′	1.50	27.78	8.94	33
	F6′	1.61	25.74	16.96	15
	F7′	4.05	0	24.28	0
	F8′	1.76	20.19	12.55	13
	F9′	7.13	15.56	20.20	0
	Extract		42.25	7.85	60

## Data Availability

A voucher specimen of each plant was deposited at the Herbarium of the School of Pharmacy at the University of Poitiers (France).

## Supplementary Materials

The following supporting information can be downloaded at: https://www.mdpi.com/article/10.3390/antibiotics11111595/s1, Table S1: List of detected and analyzed LC-MS^2^ data with bioactivity-based molecular networking for their identification and correlation with the antibiofilm activity; Figure S1: SEM approach of 24 h preformed polymicrobial biofilm C. albicans–S. aureus treated; Figure S2: Synthesis of structures putatively identified for detected ions; Figure S3: Identification of betulinic acid (BA) in Lg-AS-MeTHF extract and F4 fraction with comparison with standard: (a) HPLC-UV detection method at 210 nm; (b) MS^2^ spectrum of MS targeted method (detection MS1 at m/z 439.36); Figure S4: HPLC-UV dosage of betulinic acid (BA) from extract Lg-AS-MeTHF and active fractions F4 and F5 of L. grandiflora compared to the relative bispecies biofilm inhibition: (a) quantification table of betulinic acid with quantity per mg, final concentration per wells and their respective biofilm inhibition activity; (b) standard range of betulinic acid curve area (absorbance at 210 nm) in function of concentration.
