# Supplementary material for: Valorization of Invasive Plant Extracts against the Bispecies Biofilm Staphylococcus aureus–Candida albicans by a Bioguided Molecular Networking Screening
Source: Antibiotics (Basel). 2022 Nov 11;11(11):1595. doi: 10.3390/antibiotics11111595 (PMC9686625; doi:10.3390/antibiotics11111595)
Supplement: Supplementary file 1 [file antibiotics-11-01595-s001.zip › Supplementary Materials_Figure S1_2_3_4.pdf]

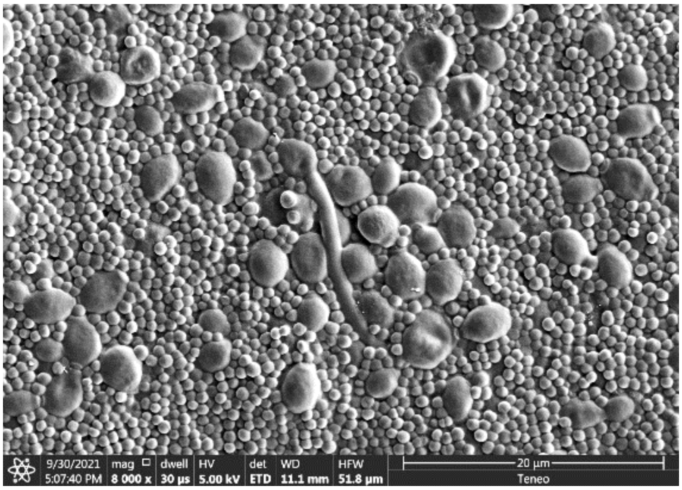

(a)

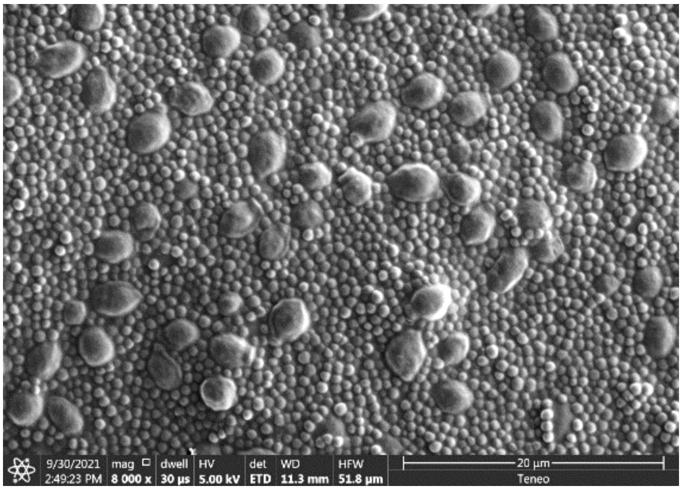

(b)

Figure S1: SEM approach of 24 h preformed polymicrobial biofilm *C. albicans*-*S. aureus* treated with DMSO 2% (a) and Lg-AS-MeTHF extract at 100 µg.mL-1 (b) for 24 h.

Supplementary Materials

Acylglycerols

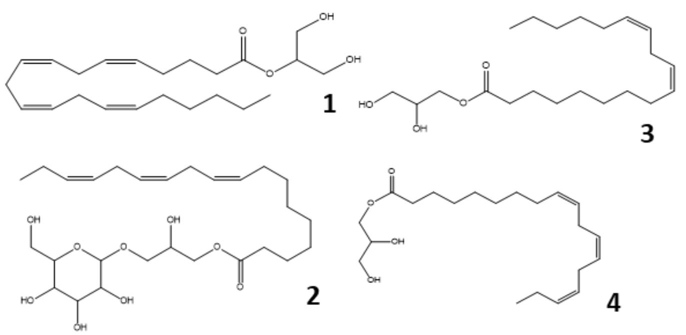

Fatty acids

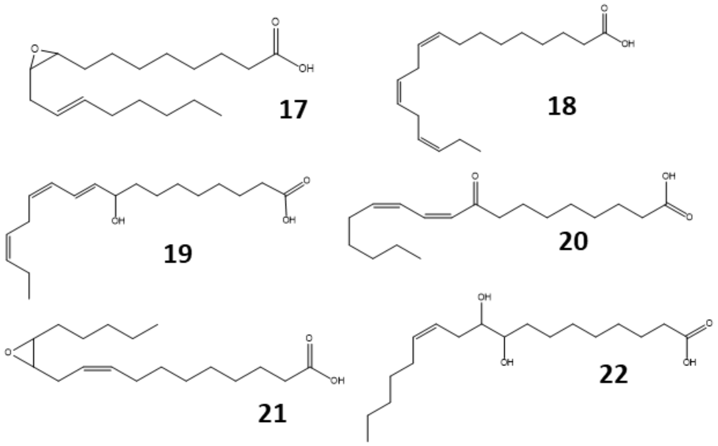

Triterpenoids

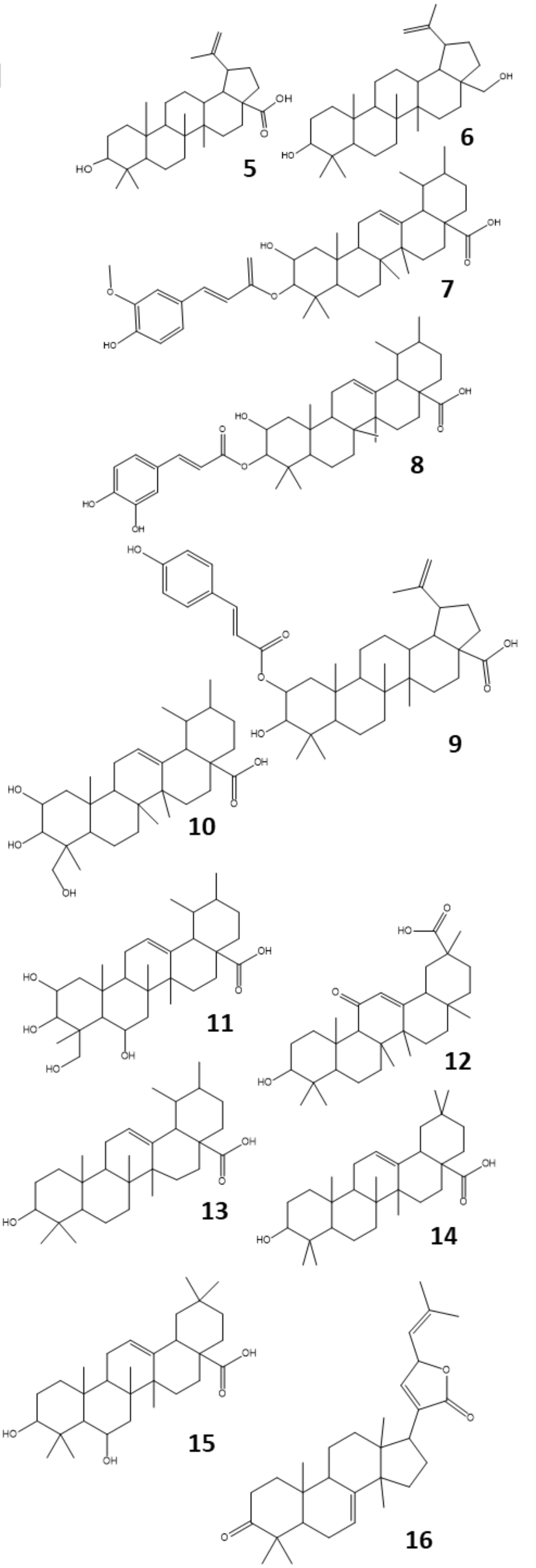

1	2-arachidonoylglycerol
2	9,12,15-octadecatrienoic acid,3-(hexopyranosyloxy)-2-hydroxypropyl ester
3	linoleoylglycerol
4	monolinolenin
5	betulinic acid
6	betulin
7	3-O-feruloyl-2-hydroxy-12-ursen-28-oic acid
8	3-caffeoyloxy-2-hydroxyurs-12-en-28-oic acid
9	2-O-p-coumaroyl alphitolic acid
10	asiatic acid
11	madecassic acid
12	enoxolone
13	ursolic acid
14	oleanolic acid
15	sumaresinolic acid
16	dysolenticin B
17	9,10-epoxy-12-octadecenoic acid
18	linolenic acid
19	9-hydroxy-10,12,15-octadecatrienoic acid
20	9-Oxo-10,12-octadecadienoic acid
21	12,13-epoxy-9-octadecenoic acid
22	9,10-dihydroxy-12-octadecenoic acid

Figure S2: Synthesis of structures putatively identified for detected ions

Supplementary Materials

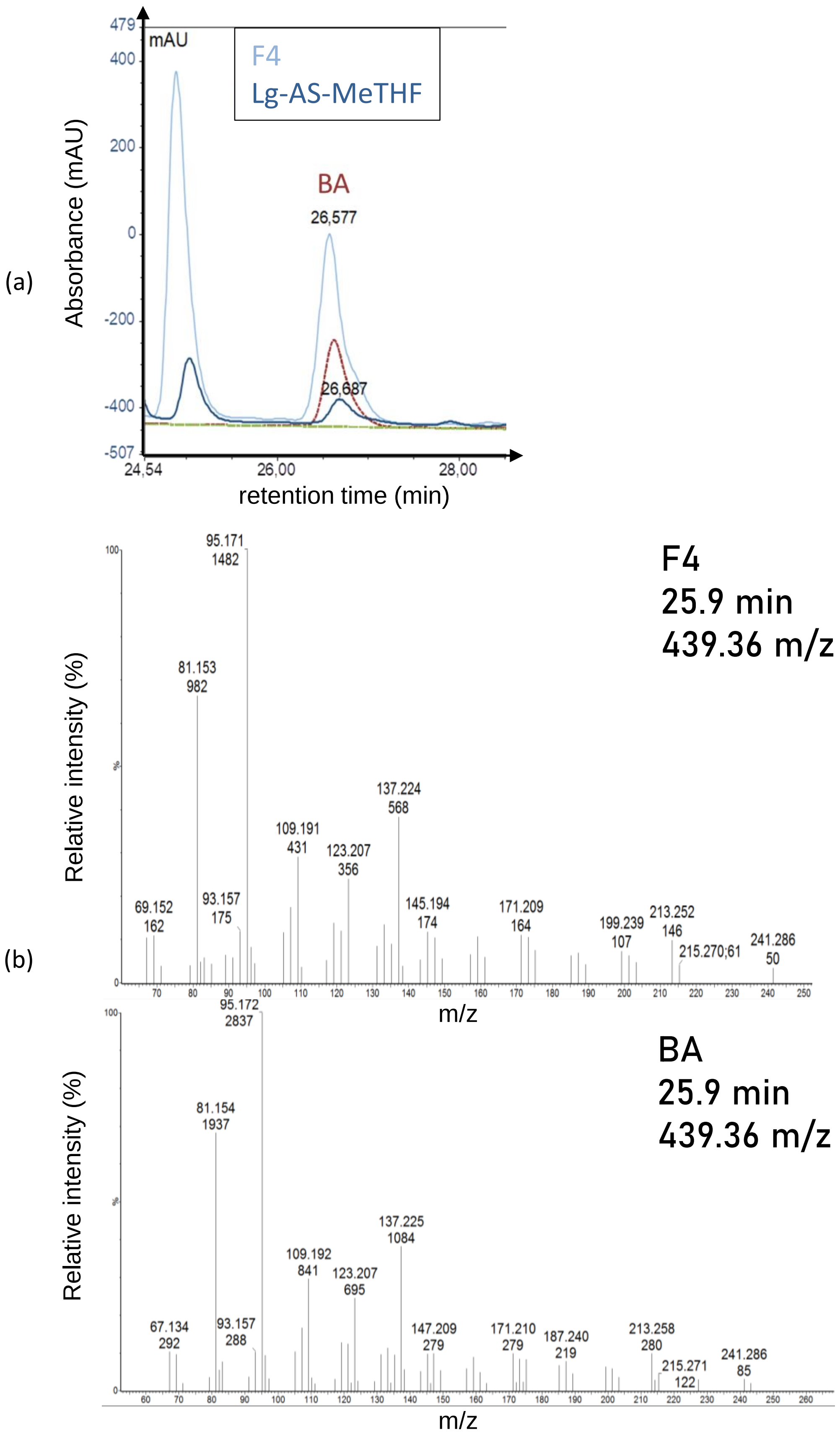

Figure S3: Identification of betulinic acid (BA) in Lg-AS-MeTHF extract and F4 fraction with comparison with standard: (a) HPLC-UV profil at 210 nm; (b) MS² spectra of MS targeted method (detection MS¹ at *m/z* 439.36).

Supplementary Materials

(a)

Sample	Peak area (mAU*min)	Quantity per mg (µg)	Final concentration per wells (µg.mL-1)	Biofilm inhibition (%)
F4 (1 mg.mL-1)	40.76	169.31 ± 1.14	8.5	64
F5 (10 mg.mL-1)	115.80	24.79 ± 1.38	1.2	50
Lg-AS-MeTHF (10 mg.mL-1)	56.38	23.73 ± 0.91	1.5	54
BA 6.25 µg.mL-1			6.25	28
BA 25 µg/mL-1			25	43

(b)

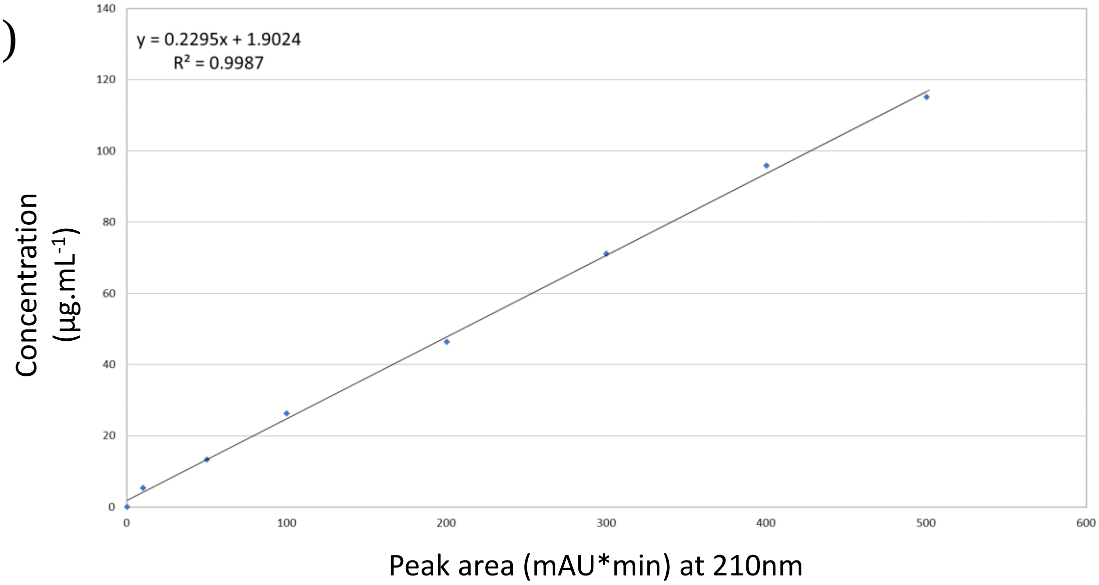

Figure S4: HPLC-UV dosage of betulinic acid (BA) from extract Lg-AS-MeTHF and active fractions F4 and F5 of *L. grandiflora*, compared to the relative bi-species biofilm inhibition: (a) quantification table of betulinic acid with quantity per mg, final concentration per wells and their respective biofilm inhibition activity; (b) standard range of betulinic acid curve area of absorbance at 210 nm in fonction of concentration. Standard calibration of BA by HPLC (210 nm)
